# Nutritional Management of Insulin Resistance in Nonalcoholic Fatty Liver Disease (NAFLD)

**DOI:** 10.3390/nu5104093

**Published:** 2013-10-11

**Authors:** Beth A. Conlon, Jeannette M. Beasley, Karin Aebersold, Sunil S. Jhangiani, Judith Wylie-Rosett

**Affiliations:** 1 Department of Epidemiology and Population Health, Albert Einstein College of Medicine, 1300 Morris Park Avenue, Bronx, NY 10461, USA; E-Mails: jeannette.beasley@einstein.yu.edu (J.M.B.); karin.aebersold@einstein.yu.edu (K.A.); judith.wylie-rosett@einstein.yu.edu (J.W.-R.); 2 Division of Gastroenterology and Clinical Nutrition, Department of Internal Medicine, Montefiore Medical Center (Wakefield), 2425 Eastchester Road, Bronx, NY 10469, USA; E-Mail: sjgimd@aol.com

**Keywords:** nonalcoholic fatty liver disease, nonalcoholic steatohepatitis, hepatic steatosis, insulin resistance, diabetes, nutrition, macronutrients, review

## Abstract

Nonalcoholic fatty liver disease (NAFLD) is an emerging global health concern. It is the most common form of chronic liver disease in Western countries, affecting both adults and children. NAFLD encompasses a broad spectrum of fatty liver disease, ranging from simple steatosis (NAFL) to nonalcoholic steatohepatitis (NASH), and is strongly associated with obesity, insulin resistance, and dyslipidemia. First-line therapy for NAFLD includes weight loss achieved through diet and physical activity. However, there is a lack of evidenced-based dietary recommendations. The American Diabetes Association’s (ADA) recommendations that aim to reduce the risk of diabetes and cardiovascular disease may also be applicable to the NAFLD population. The objectives of this review are to: (1) provide an overview of NAFLD in the context of insulin resistance, and (2) provide a rationale for applying relevant aspects of the ADA recommendations to the nutritional management of NAFLD.

## 1. Introduction

**Figure 1:**
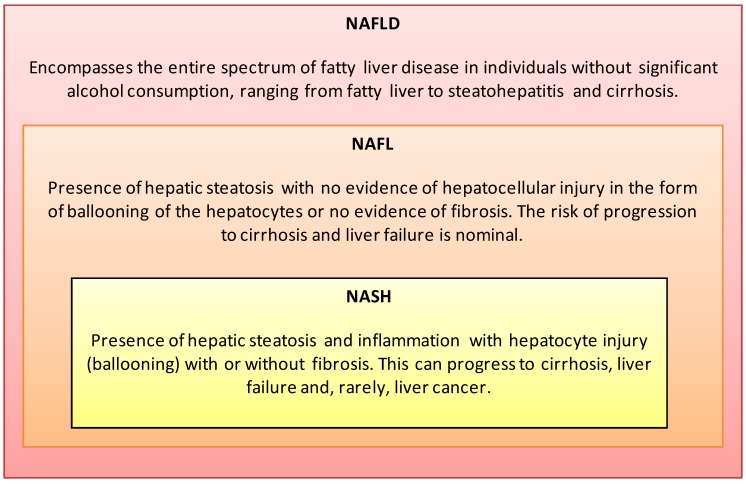
Nonalcoholic Fatty Liver Disease (NAFLD) is histologically categorized into Nonalcoholic Fatty Liver (NAFL) and Nonalcoholic Steatohepatitis (NASH) [1,7,11].

Non-Alcoholic Fatty Liver Disease (NAFLD) is an increasing global health concern, with an estimated prevalence of 20%–30% in Western countries and 15% in Asian countries [1]. The spectrum of NAFLD ranges from simple steatosis (NAFL) to nonalcoholic steatohepatitis (NASH) and cirrhosis (Figure 1). Approximately 3%–5% of patients with hepatic steatosis develop NASH, which may progress to end-stage liver disease or hepatocellular carcinoma [2]. NASH is projected to surpass hepatitis C virus (HCV) as the leading indication for liver transplantation in the United States over the next 20 years [3].

NAFLD is tightly linked with obesity and is regarded as the hepatic manifestation of the metabolic syndrome. Prevalence of NAFLD among individuals with type 2 diabetes is estimated to be 65%–70%, which is more than twice the prevalence among people without diabetes [4,5]. Furthermore, NAFLD may be an independent predictor of future risk of cardiovascular events in the diabetic population [6]. First-line therapy for NAFLD includes weight loss achieved through diet and physical activity [7], but there is a lack of evidence-based nutrition recommendations to guide dietary intervention. The American Diabetes Association (ADA) [8,9,10] provides evidence-based nutrition recommendations that aim to reduce the risk of diabetes and cardiovascular disease (CVD), with goals that include regulating blood glucose and insulin levels, and improving lipid and lipoprotein profiles. The ADA recommendations are potentially applicable to the NAFLD population, given the high comorbidity rates shared among hepatic steatosis, insulin resistance, and CVD. The objectives of this review are to: (1) Provide an overview of NAFLD in the context of insulin resistance, and (2) build upon this information to provide a rationale for applying relevant aspects of the ADA recommendations [8,9,10] to the nutritional management of NAFLD.

## 2. Definition and Risk Factors

In 2012, the American Association for the Study of Liver Diseases (AASLD), in collaboration with the American College of Gastroenterology and the American Gastroenterological Association, published the first evidence-based practice guidelines (AASLD Guidelines) for the diagnosis and management of NAFLD in both adults and children [7]. Prior to the AASLD Guideline, Ratziu and colleagues published a position statement summarizing the proceedings of the 2009 European Association for the Study of The Liver’s (EASL) conference on NAFLD/NASH, and Sanyal and colleagues [12] summarized the consensus of a 2009 AASLD research workshop on endpoints and clinical trial design for NASH. The AASLD Guideline [7] definitions are provided in Figure 1.

According to the AASLD Guidelines [7], liver biopsy is the gold standard for the diagnosis of NAFLD, however imaging (e.g., ultrasonography) is more commonly used because of increased health risks and expenditures associated with liver biopsies. Criteria for diagnosis include hepatic steatosis by imaging or histology, exclusion of significant alcohol consumption (≥20 g alcohol/day in women and ≥30 g alcohol/day in men), and no existing hereditary disorders or competing etiologies for hepatic steatosis [7,12]. NAFLD is categorized as primary or secondary. Primary is associated with insulin resistance and metabolic derangements, and secondary is associated with non-insulin related conditions, as described elsewhere [13]. However, given that the prevalence of adult overweight and obesity exceeds 50% in many developed countries [14], it is not uncommon to observe primary NAFLD in the presence of other chronic liver diseases, such as alcoholic liver disease and chronic HCV [15].

The risk of advanced liver disease in NAFLD patients increases as the number of metabolic comorbidities increase [16]. Available data indicate that the prevalence of NAFLD likely exceeds 65% in type 2 diabetic populations and greater than 90% in morbidly obese populations [6,17]. NAFLD shares common risk factors with type 2 diabetes and other diseases influenced by lifestyle (Table 1), making lifestyle intervention a rational treatment for NAFLD. The prevalence of NASH is difficult to establish because a liver biopsy is required for diagnosis. More than 30% of morbidly obese patients may have NASH and 12%–25% have fibrosis [17,18,19]. Diabetes and insulin resistance may be more important predictors of NASH and fibrosis than BMI [20,21]. Moreover, due to the high prevalence of obesity among children [22], type 2 diabetes and related metabolic disorders are appearing in youth. An estimated 9.6% of the general pediatric population and 38% of the obese pediatric population in the United States are affected by NAFLD [23]. Pediatric NAFLD is likely to progress into adulthood, making prevention, early detection, and treatment an important issue across the lifecycle.

**Table 1:** Clinical and lifestyle risk factors associated with NAFLD [7,11].

**Clinical Risk Factors**
• Obesity
• Insulin resistance ^a^
• Type 2 diabetes ^a,b^
• Metabolic Syndrome ^c,d^ (↑ central adiposity, dyslipidemia, hypertriglyceridemia, hypertension, ↑ fasting glucose)
• Cardiovascular disease
• Endocrine (polycystic ovary syndrome, hypothyroidism, hypopituitarism, hypogonadism)
• Gallbladder disease
• Pancreato-duodenal resection
• Obstructive sleep apnea
• Starvation/malnutrition
**Lifestyle Risk Factors**
• Demographics (↑ age, first degree relatives of individuals with obesity or diabetes, sex ^e^, race ^f^)
• Western countries
• Western diet (↑ calories, ↑ saturated fat, ↑ trans fat, ↓ intake of *n*-3 fatty acids, vitamin D, and fruits and vegetables)
• Physical inactivity

Abbreviations: ↑ = increase in; ↓ = indicates decrease in. ^a^ Independent predictors of liver-related mortality in NAFLD [24]. ^b^ Type 1 diabetics have increased prevalence of NAFLD, based on liver imaging with limited histological evidence [25]. ^c^ Independent predictor of NASH risk [20]. ^d^ Metabolic syndrome lacks consistent definition [26,27]. AASLD Guidelines [7] cite Adult Treatment Panel III (ATP III) of the National Cholesterol Education Program [28]. ^e^ Men may have greater risk than women [29,30]. ^f^ Data from the U.S. indicate Hispanics and whites are at greater risk than blacks [29,31].

## 3. Pathophysiology

**Figure 2:**
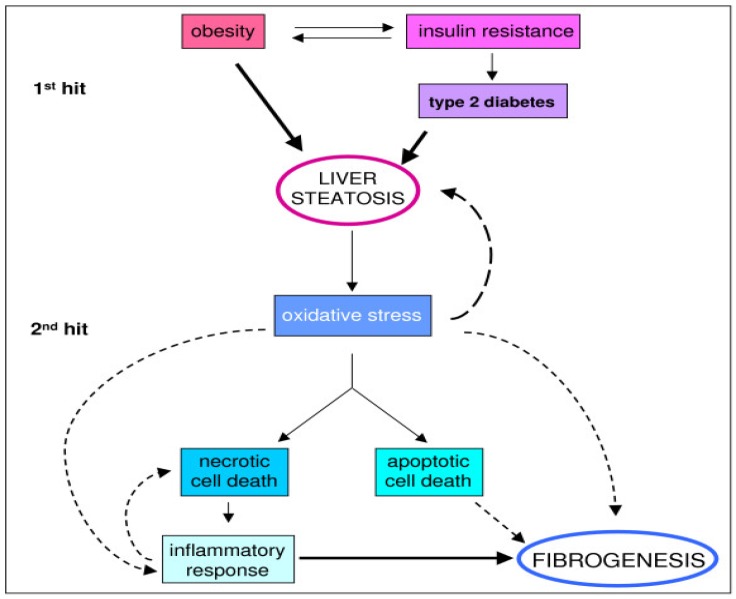
The two-hit hypothesis of NAFLD progression [34]. Reproduced from [35] (Copyright © 2008 Novo *et al.*; licensee BioMed Central Ltd.).

NAFLD is a multifactorial disease with complex pathophysiology. Clinical hallmarks of NAFLD include obesity, insulin resistance, and dyslipidemia [32]. Adiposity is associated with a pro-inflammatory state, mediated by hormones and cytokines, such as tumor necrosis factor and interleukin-1. Hepatic lipid dysregulation, oxidative stress, and pro-inflammatory cytokines interact synergistically to promote hepatic fat accumulation over time [33]. Fatty infiltration of the liver results from an imbalance between hepatic lipid accumulation (from accelerated free fatty acid influx and *de novo* lipid synthesis) and hepatic lipid clearance (free fatty acid oxidation (FAO) and very low-density lipoprotein excretion). Accumulation of intracellular lipids is one proposed mechanism of insulin resistance, although whether insulin resistance is a cause or consequence of lipid accumulation remains debated. Day and colleagues [34] originally described this phenomenon in their “two-hit hypothesis” of the pathogenesis of NAFLD (Figure 2).

The first hit is metabolically driven, involving insulin resistance and lipid accumulation in hepatocytes [35]. Liver steatosis is associated with both hepatic and adipose tissue insulin resistance, as well as reduced systemic insulin sensitivity. Insulin resistance increases the rate of peripheral adipose tissue lipolysis, creating an increased flux of free fatty acids into the liver. Hyperinsulinemia and hyperglycemia promote lipid accumulation in hepatocytes by stimulating *de novo* lipogenesis while inhibiting FAO and lipid exportation in the liver, by mechanisms described elsewhere [32,36]. In the context of insulin resistance, a depletion of hepatic *n*-3 polyunstaurated fatty acids (PUFAs) has been reported [37]. *n*-3 PUFAs may play a critical role in regulating the metabolic switch from anabolism (lipogenesis) to catabolism (FAO) by activating peroxisome proliferator-activated receptor alpha (PPARα), a positive regulator of FAO [38,39]. The second hit occurs when lipid accumulation becomes toxic and induces a stress response in the liver, involving inflammation and oxidative stress. This can result in necrotic cell death, programmed (apoptotic) cell death, and lead to fibrosis and NASH.

NASH occurs in a small subset of patients, which suggests that additional factors play a role. The pathogenesis of NASH is marked by increased inflammation, cytokines, and oxidative stress. A study of mice fed a high-fat diet for 8 or 12 weeks showed increased expression in the transcription factor, nuclear factor erythroid 2-related factor 2, a key regulator of redox homeostasis in cells, concomitantly with elevated liver index, alanine aminotransferase (ALT), cholesterol, and glucose [40]. Ibdah and colleagues [41] reported that aging mice with a heterozygous mutation for mitochondrial tri-functional protein, a critical enzyme in long-chain (13–21 carbon atoms) FAO, developed concomitant NAFLD and insulin resistance with higher levels of oxidative stress, as indicated by increased expression of cytochrome P-450 2E1 and decreased expression of glutathione. In a clinical study of adults (*n* = 82) with biopsy-proven NAFLD, altered secretion of obesity-related cytokines, such as adiponectin, leptin, and ghrelin were associated with more advanced NAFLD [42]. Antioxidant therapy for the treatment of NAFLD, including NASH, has the potential to ameliorate the oxidative stress and damage that promotes disease progression [43].

Collectively, these findings demonstrate the complex pathogenesis of NAFLD, and highlight areas for potential nutrition intervention. Weight loss decreases adipose tissue, thereby reducing both peripheral adipose tissue and hepatic insulin resistance (Figure 2). The AASLD Guidelines [7] recommend a weight loss of at least 3%–5% body weight to improve steatosis, and a weight loss of up to 10% may be needed to improve necroinflammation (Table 2).

Intensive lifestyle intervention programs may be the most effective strategy for achieving these recommendations. Suzuki and colleagues [44,45] reported that a 5% weight reduction was sufficient for decreasing serum ALT, and the Look AHEAD trial [46] showed that a 12-month intensive lifestyle intervention program (average 8% weight loss) reduced steatosis, measured by proton magnetic resonance spectroscopy, and hemoglobin A1c (HbA1c) in overweight and obese adults with type 2 diabetes. The ADA recommends a 5%–10% [8,9,10] weight loss for the prevention and management of diabetes and CVD, consistent with the AASLD Guidelines [7] for NAFLD. However, the ADA recommendations [8,9,10] provide further guidance for medical nutrition therapy (MNT), with considerations given to macronutrients and micronutrients, including antioxidants and fatty acids [47]. MNT is an evidenced-based practice that delivers nutritional diagnosis, therapy, and counseling services for the purpose of disease management [48]. In the following section, we provide rationale for using the ADA recommendations [8,9,10] to guide dietary intervention in the NAFLD population and identify gaps for future research and clinical opportunities.

## 4. Medical Nutrition Therapy

A key difference between the ADA recommendations [8,9,10], AASLD Guidelines [7], and Dietary Guidelines for Americans (DGAs) [49] is that the DGAs are targeted to the general population rather than populations at high metabolic risk (Table 2). Similarly, existing obesity guidelines such as those established by the National Heart, Lung, and Blood Institute [50], emphasize delivery of patient care rather than individual management of metabolic risks. Thus, the ADA recommendations [8,9,10] are reasonable evidenced-based guidelines for the nutritional management of NAFLD and its associated comorbidities. Table 2 provides a summary of and comparison between the ADA recommendations [8,9,10], AASLD Guidelines [7], DGAs [49], and a proposed dietary framework from a recent review on the role of diet and nutrient composition in NAFLD by McCarthy and Rinella [47].

**Table 2:** Nutrition Guidelines and Recommendations for the General Population, Diabetes, and NAFLD.

Organizations	USDHHS, USDA [49]	ADA [8,9,10]	AASLD, ACG, AGA [7]	McCarthy and Rinella [47]
Document Type	Evidenced-based Guidelines	Evidenced-based Guidelines	Evidenced-based Guidelines	Professional Review
Population	General U.S. population.	Diabetes/Prediabetes	NAFLD/NASH	NAFLD/NASH
Weight Loss	Consume fewer calories than expended. This can be achieved over time by eating fewer calories, being more physically active, or, best of all, a combination of the two.	Either low-carbohydrate or low-fat calorie-restricted diets may be effective short-term (up to one year).	3%–5% of body weight appears to improve steatosis; up to 10% weight loss may be needed to improve necroinflammation. May be achieved either by hypocaloric diet alone or with increased physical activity.	Initial goal: 5%–10% body weight lost over one year. Long-term goal: ideal body weight, maintenance of weight loss.
Energy (calories)	Balance calories to maintain weight.	Hypocaloric diet for weight loss based on individual needs.	Hypocaloric diet alone or with physical activity to promote weight loss.	1200–1500 cal/day.
Carbohydrate	Limit the consumption of foods that contain refined grains, especially refined grain foods that contain solid fats, added sugars, and sodium. Reduce the intake of calories from added sugars.	A dietary pattern that includes carbohydrate from fruits, vegetables, whole grains, legumes, and low-fat milk is encouraged for good health. Whole grains should be one-half of total grain intake. Fiber intake same as general population (14 g fiber/1000 kcal).	Not specified.	≥50% whole grain; avoid high-fructose corn syrup.
Protein	10%–35% total calories.	Insufficient evidence to suggest that usual protein intake (15%–20% of energy) should be modified.	Not specified.	Lean animal- or vegetable-based protein.
Total Fat	20%–35% total calories.	Varies with diet; low-fat or low-carbohydrate diet for weight loss.	Not specified.	<35% of total calories.
Saturated Fat	<10% of total calories	<7% of total calories.	Not specified.	<7% of total calories
Trans Fat	As minimal as possible.	As minimal as possible.	Not specified.	As minimal as possible.
Unsaturated Fatty Acids	Replace saturated fats with MUFA and PUFA.	Two or more servings of fatty fish per week (with the exception of commercially fried fish filets).	Premature to recommend; may be considered as the first line agents to treat hypertriglyceridemia in patients with NAFLD.	Fish oil 1 gram/day (eicosapentaenoic + docosahexaenoic acids). Up to 25% MUFA.
Cholesterol	<300 mg/day.	<200 mg/day.	Not specified.	Not specified.
Micronutrients	Meet the Recommended Dietary Allowance or Adequate Intake.	No clear evidence of benefit from vitamin or mineral supplementation in people with diabetes (compared to the general population) who do not have underlying deficiencies.	Vitamin E 800 IU/day in non-diabetic adults with biopsy-proven NASH. Not recommended to treat NASH in diabetic patients, NASH cirrhosis, or cryptogenic cirrhosis.	Vitamin E 800 IU/day.
Sodium	<2300 mg/day general population; <1500 mg/day if ≥51 years of age, African American or have hypertension, diabetes, or chronic kidney disease.	<2300 mg/day in normotensive and hypertensive individuals; <2000 mg/day in diabetics and patients with symptomatic heart failure.	Not specified.	Not specified.
Alcohol	If consumed, consume in moderation (one drink/day or less for women and two drinks/day or less for men).	If consumed, consume in moderation (one drink/day or less for women and two drinks/day or less for men).	Patients with NAFLD should avoid heavy amounts of alcohol (3 drinks/day for women, 4 drinks/day for men).	Not specified.

Abbreviations: USDHHS = United States Department of Health and Human Services; USDA = United States Department of Agriculture; ADA = American Diabetes Association; AASLD = American Association for the Study of Liver Diseases; ACG = American College of Gastroenterology; AGA = American Gastroenterological Association; NAFLD = Nonalcoholic Fatty Liver Disease; NASH = Nonalcoholic Steatohepatitis; MUFA = monounsaturated fatty acid; mg = milligrams; IU = international units.

### 4.1. Carbohydrates: Quantity and Quality

The ADA does not recommend a single macronutrient distribution for all individuals with diabetes [8,9,10]. A dietary intake pattern that emphasizes whole grains, fruits, vegetables, legumes, and low-fat dairy is recommended for disease prevention and weight maintenance (Table 2). A hypocaloric, low-carbohydrate diet (<40% carbohydrate) or low-fat diet (<30% fat) are recommended for weight loss. Low-carbohydrate diets may result in greater short-term (<6 months) weight loss than a low-fat diet, but both diets have similar long-term (>1 year) health benefits [51,52,53,54].

The quality or type of carbohydrate is likely to influence the development of NAFLD [47]. Excessive intake of simple or refined carbohydrates has been associated with obesity and insulin resistance in adults and children. A Western dietary pattern [55,56], which includes soft drinks, candies, and simple or refined carbohydrates, is a risk factor for NAFLD and is typical of a high glycemic index (GI) dietary pattern. The glycemic index is a quantitative measure of a food’s carbohydrate content through its glycemic effects [57]. Low-GI foods elicit a low postprandial glucose response and their regular consumption has been associated with reduced risk of diabetes and CVD [58,59,60]. Valtueña *et al.* [61] demonstrated a positive relationship between higher quartiles of dietary GI with higher grades of hepatic steatosis in a population of 247 apparently healthy adults. Because dietary GI and total carbohydrate intake are distinct measurements, the ADA encourages clinicians and researchers to consider the use of the GI and glycemic load, as it may provide a modest additional benefit over that observed when total carbohydrate is considered alone (Table 2) [8].The low-fat dietary pattern recommended by the ADA typically features a low GI dietary pattern.

However, when the percentage of dietary carbohydrate is changed, the amount of fat and protein invariably change. These variations in macronutrient distributions make it difficult to attribute the effects of dietary changes to a single macronutrient (carbohydrate, protein, or fat), and to apply a universal definition to “low-carbohydrate” and “high-carbohydrate” diets, making it difficult to compare studies [62]. With these limitations in mind [62], several studies that have examined the effects of low-carbohydrate or low-fat diets on NAFLD are highlighted.

In a cross-sectional analysis, Kang *et al.* [21] observed that patients with metabolic syndrome consumed significantly more dietary carbohydrates (51% *vs.* 45% total energy) and less dietary fat (34% *vs.* 40% total energy) than patients without metabolic syndrome. Those with metabolic syndrome also had significantly higher homeostatic model assessment index, a quantitative measure of insulin resistance, and greater histological severity of NAFLD. Ryan *et al.* [63] randomized obese, insulin-resistant participants to receive a hypocaloric diet containing moderately low-carbohydrate (40%–45%) intake, or a hypocaloric diet containing moderate-carbohydrate (60%) intake, for 16-weeks. The moderately-low carbohydrate diet group had significantly greater decreases in serum ALT and circulating insulin, indicating the low-carbohydrate diet may be more effective for short-term health improvements. The Fatty Liver Ancillary Study of the Look AHEAD (Action for Health in Diabetes) trial [46] observed individuals randomized to receive 1-year of treatment to an intensive lifestyle intervention (ILI) group (moderate caloric restriction, <30% fat of total energy, increased physical activity, weight loss goal of 7% body weight) or diabetes support and education (DSE) control group. The ILI group experienced significant reductions in hepatic steatosis and HbA1c, but not in AST or ALT. At 12-months, 26% of the DSE participants *versus* 3% of the ILI participants without NAFLD at baseline developed NAFLD. Haufe and colleagues [64] randomized 102 overweight and obese individuals to a 6-month dietary intervention consisting of either low-carbohydrate or low-fat hypocaloric diets, and observed improvements in intrahepatic lipid content in both groups that did not significantly differ. However, subjects with high baseline intrahepatic lipids (>5.56%) lost approximately 7-fold more intrahepatic lipid than those with lower baseline values. Collectively, these studies do not discern significant differences between low-carbohydrate and low-fat diets for periods of greater than 6-months, which is consistent with observations in diabetic populations, as summarized by ADA reports [8,9,10].

However, it is likely that a very low-fat diet high in simple sugars may stimulate de novo fatty acid synthesis in the liver, independent of BMI and insulin [65,66]. McCarthy and Rinella [47] reported that increased intake of high fructose corn syrup (HFCS) is likely attributed to an increased risk of NAFLD [67,68]. Adults and children with NAFLD may consume 2–3 times more HFCS than non-NAFLD patients [67], and the lipid metabolism of children with NAFLD may be more sensitive to the metabolic effects of HFCS than children without NAFLD [69]. Decreasing or avoiding consumption of HFCS may be important in the nutrition intervention for the NAFLD population, particularly in insulin resistant patients with poor glycemic control. Similarly, the ADA recommendations [8,9,10] encourage individuals to limit consumption of simple or refined carbohydrates, and increase consumption of complex carbohydrates, such as those found in whole grains, legumes, fruits, and vegetables (Table 2).

While there is no clear consensus on the ideal macronutrient distribution of carbohydrates, a low-fat/high-GI diet or reduced carbohydrate diet may facilitate short-term (<1 year) weight loss. Future investigations are warranted, particularly in long-term (>1 year) randomized clinical trials (RCTs) to evaluate the effects of varying carbohydrate distributions, and study designs that control for the potential effects of weight loss. It is important to note that restricting total carbohydrate to less than 130 g/day, is not recommended for individuals with diabetes [8].

### 4.2. Total Fat and Fatty Acids

A Western-style dietary pattern is a risk factor for NAFLD, and is typically characterized by a high intake of saturated fatty acids (SFAs) with low amounts of *n*-3 PUFAs (eicosapentaenoic acid 20:5 [EPA], and docosahexaenoic acid 22:6 [DHA]) and monounsaturated fatty acids (MUFAs) [70]. High amounts of SFAs and low amounts of *n*-3 PUFA consumption are associated with oxidative stress, decreased FAO, and depletion of PUFAs in the liver [70]. Similar to *n*-3 PUFAs, diets low in MUFAs are associated with decreased levels of PPARα particularly in the context of insulin resistance [71]. Dietary patterns rich in *n*-3 PUFAs and MUFAs may play an important role in preventing and treating NAFLD, as well as improving lipid metabolism in insulin resistant individuals [9,72].

Bozetto *et al.* [73] published the first randomized intervention study to evaluate the effects of dietary strategies with and without exercise on liver fat content, independent of weight loss. The study evaluated hepatic fat content by proton nuclear magnetic resonance spectroscopy in 37 men and 8 women (ages 35–70 years) before and after randomization to one of four intervention groups (Table 3). The dietary interventions were based on two dietary strategies recommended by the ADA: (1) a high-carbohydrate (>50%), high fiber, and low GI diet, and (2) a reduced carbohydrate (40%–45%) diet enriched with MUFAs. The diets were isocaloric and participants maintained weight throughout the study duration.

**Table 3:** Summary of dietary intervention groups in study by Bozzetto *et al.* [73].

Group	CHO%	Fat%	MUFA%	Fiber/1000 kcal	GI	Supervised Exercise
CHO/fiber	52	30	16	28	60	No
CHO/fiber + Exercise	52	30	16	28	60	Yes
MUFA	40	42	28	10	95	No
MUFA + Exercise	40	42	28	10	95	Yes

Abbreviations: CHO = carbohydrate; MUFA = monounsaturated fatty acid, as percentage of total; kcal = kilocalories; GI = glycemic index; Exercise = supervised exercise, 45-min 2×/week.

After 8 weeks of intervention, hepatic fat content significantly decreased in the MUFA (−29%) and MUFA + exercise (−25%) groups, compared to the CHO/fiber (−4%) and CHO/fiber +exercise groups (−6%). There were no significant effects for exercise training or diet-exercise interaction. The CHO/fiber and high-MUFA groups did not differ in SFAs (7%), but varied in total fat and MUFAs. Westerbacka *et al.* [74] was the only other study to measure diet-induced changes in hepatic fat content in the absence of weight loss. In a group of 10, non-diabetic overweight women, a high-fat diet (56% total energy) promoted increased hepatic steatosis and serum insulin resistance, in comparison to a low-fat diet (16% total energy) [74]. However, the high-fat group consumed significantly more saturated fat, which likely confounded the association between the high-fat diet and hepatic steatosis.

The ADA Systematic Review [9] identified one RCT that compared saturated fatty acid (SFA) intake with MUFA intake while total fat remained the same, but did not find significant differences in postprandial glucose or insulin response [9,75]. This led to question whether decreasing SFA and/or increasing MUFA exerts beneficial effects on postprandial triglyceride levels. McCarthy and Rinella’s review identified evidence to support negative effects of high (>10%) and low (<6%) dietary SFA consumption, and suggested that a range of SFA between 6% and 10% may be most beneficial to patients with NAFLD (Table 2), with an intake of MUFA up to 25%, and increased intake of *n*-3 PUFAs. The ADA and DGAs (Table 2) similarly encourage increased consumption of MUFAs and *n*-3 PUFAs, and decreased consumption of SFAs and trans fatty acids. The AASLD Guidelines [7] acknowledge that unsaturated fatty acids may be beneficial for NAFLD patients with hypertriglyceridemia.

The Mediterranean-style diet is touted for its associations with improved cardiovascular health [76], and is low in carbohydrates with high amounts of MUFA, vegetables, fruits, legumes, olive oil/nuts (MUFA), and fish (*n*-3 PUFAs), with moderate amounts of wine and minimal amounts of SFAs. There is not a standalone definition for the Mediterranean-style eating pattern, but rather it is typically reflective of geographical and cultural practices of inhabitants of the Mediterranean region. Perez-Guisado and Munos-Serrano [77] administered the Spanish Ketogenic Mediterranean Diet (≤30 g of carbohydrates/day from green vegetables and salad, at least 30 mL of olive oil, 200–400 mL of red wine, no protein or calorie restriction) to 14 obese men over a 12-week period [78], and observed significant improvements in body weight, low-density lipoprotein cholesterol, metabolic syndrome parameters, and degree of hepatic steatosis. These findings corroborated the results of a prior study that tested the efficacy and safety of the diet [77]. While diets low in carbohydrates and high in MUFAs are evidenced to improve outcomes in NAFLD patients, it is difficult to discern whether the effects are attributed to the high MUFA, reduced carbohydrate intake (with possible ketogenesis), or both. Future studies should consider a comparison group to help answer this question, such as a moderate- to high-carbohydrate enriched MUFA group.

Moreover, the contribution of wine and *n*-3 PUFAs to the perceived health benefits of low-carbohydrate, enriched MUFA dietary patterns should be considered. Data from the National Health and Nutrition Examination Survey indicated participants who consumed up to 10 g of wine per day (*n* = 945) were half (OR = 0.51; 95% CI 0.33, 0.79) as likely to meet the criteria for suspected NAFLD (defined by elevated liver function tests) [79]. The ADA Systematic Review [9] concluded that individual components of the Mediterranean-style diet, including wine and high MUFA/olive oil, may not have independent effects on glycemic control, but may independently benefit cardiovascular risk factors.

The effect of *n*-3 PUFA supplementation on improving insulin resistance remains equivocal; however, eating fatty fish rich in *n*-3 PUFAs at least twice a week remains a key strategy for diabetes management. *n*-3 PUFA supplements have been associated with increases in fasting plasma glucose, but they are also associated with reductions in HbA1c and CVD risk factors [9,80,81]. Several studies have demonstrated effectiveness of *n*-3 PUFAs in the treatment of NAFLD. DHA supplementation (250–500 mg/day) significantly improved liver steatosis and insulin sensitivity in an RCT in children (*n* = 60) [82]. *n*-3 PUFA supplementation in adult NAFLD patients similarly demonstrated improvements in NAFLD, glycemic control, and lipid profiles at 6-months (2–6 g/day) [83,84] and 12-months (1 g/day) [85]. One-year of dietary supplementation with olive oil enriched with *n*-3 PUFA also showed significant improvements in liver enzymes and triglycerides, with reported increases in adiponectin levels [86]. In an evaluation of the efficacy of *n*-3 PUFAs as a hypolipidemic agent in NAFLD patients with hypertriglyceridemia, 6-months of *n*-3 PUFA (15 mL/day) supplementation resolved fatty liver in 35% of patients [87]. EPA has similarly been shown to improve NASH, likely due to its antioxidative and anti-inflammatory properties [88]. These studies demonstrate an important role of *n*-3 PUFAs in the amelioration and possible reversal of NAFLD. Clinical trials examining the efficacy and dose-response of *n*-3 PUFA treatment, as well as the role of *n*-3 PUFAs in prevention are needed.

Although limited, research on dietary fat and fatty acids in patients with NAFLD trends favorably towards a moderate to low-carbohydrate diet with increased MUFA and *n*-3 PUFAs. Available data in the NAFLD population appears consistent with the ADA recommendations [8,9,10] and American Heart Association’s diet and lifestyle recommendations [72]. Future research to support the work of Bozetto *et al.* [73] and further investigate the effects of Mediterranean-style diets and *n*-3 PUFA consumption or supplementation on hepatic steatosis is needed.

### 4.3. Protein

The AASLD Guidelines [7] do not specifically address protein intake, and the ADA [8,9,10] recommends a protein intake similar to the general population, based on the recommended dietary allowances (RDA) and dietary reference intakes (Table 2). However, similar to carbohydrates, the definition of protein varies widely in the literature. High-protein diets have been classified as anywhere from 27% to 68% of daily energy intake or about 90 to almost 300 g/day in absolute amounts [89]. When comparing a high-protein (30% of total calories), low-carbohydrate diet (20% of total calories) to a moderate-carbohydrate (55% of total calories), low-protein (15% of total calories) diet among individuals with Type 2 Diabetes, Gannon and Nuttall [90,91] reported that the high-protein, low-carbohydrate diet reduced fasting plasma glucose, 24-h glucose and HbA1C. The ADA has suggested, in the absence of diabetic kidney disease, higher protein eating patterns (30% of calories) may or may not improve HbA1c; however, they appear to improve one or more CVD risk measures [9]. For individuals with diabetic kidney disease in the presence of either micro- or macroalbuminuria, reducing the amount of protein from normal levels does not appear to alter glycemic measures, CVD risk measures, or the course of renal deterioration [92]. Because protein content changes with carbohydrate and fat, protein changes are typically observed in the context of low-carbohydrate *versus* moderate- to high-carbohydrate diets, as discussed previously.

Haufe and colleagues [64] randomized 170 overweight and obese individuals to a 6-month dietary intervention with hypocaloric diets (30% reduction from usual caloric intake, to a minimum of 1200 cal/day) consisting of low-carbohydrate (≤90 g/day), moderate-protein (0.8 g/kg/day) or reduced-fat (<20%), moderate-protein (0.8 g/kg/day) intake with the remaining calorie content from carbohydrates. Of 102 subjects that completed the dietary interventions, decreased body weight and improvements in body composition, visceral fat, and intrahepatic lipid content were observed in both groups. There were not significant changes in intrahepatic lipid content, measured by fat spectroscopy, but subjects with high baseline intrahepatic lipids (>5.56%) lost approximately 7-fold more intrahepatic lipid than those with lower baseline values. These results suggest that in the longer term of 6-months, both low-carbohydrate/high-protein and low-fat diets may be beneficial. However, there is insufficient evidence to provide recommendations on protein intake specific for NAFLD, and following the ADA [8,9,10] and DGAs [49] appear appropriate (Table 2).

### 4.4. Micronutrients

The ADA Recommendations [8,10] do not support a vitamin or mineral intake beyond what is recommended for the general population. Similarly, a recent Cochrane review concluded that there is insufficient evidence for or against the use of supplementation in patients with NAFLD [93]. However, antioxidants may benefit the patient by protecting against oxidative stress.

The AASLD Guidelines [7] recommend a vitamin E intake of 800 international units (IU) per day as first-line therapy for non-diabetic adults with biopsy-proven NASH (Table 2), and benefits have also been reported in children [94,95]. However, limited data exists on the effect of vitamin E in the general NAFLD population. In a 12-month, double-blind placebo study in a pediatric population (*n* = 90), lifestyle intervention plus supplementation with vitamin E (600 IU/day) and ascorbic acid (500 mg/day) resulted in significant improvement in liver function and glucose metabolism, but did not significantly differ from the lifestyle intervention only arm [96]. In a subsequent 24-month RCT [97], vitamin E (600 IU/day) plus ascorbic acid (500 IU/day) did not have an effect on increasing the efficacy of lifestyle intervention, but increased physical activity and weight loss were strongly associated with improvements. Vajro *et al.* [98] conducted a randomized study on vitamin E and hypocaloric dietary intervention and reported that children who adhered only to vitamin E had complete normalization of transaminase levels, without significant changes in weight status, suggesting independent effects of vitamin E. Two studies comparing the effects of therapeutic intervention for NAFLD supplemented with vitamin E or metformin in children and adolescents [99], and adults [100], did not find evidence of superiority in the vitamin E group. The high-MUFA diet group in the study by Bozetto *et al.* [73] contained higher amounts of vitamin E [73], questioning the benefits of dietary *versus* supplemental forms of vitamin E. The use of vitamin E supplementation is controversial because it has been associated with increased risk of certain types of cancers, bleeding, and hemorrhagic stroke [101], thus, future investigations into its safety and efficacy are warranted.

Reduced intake of vitamin D is common in the Western diet. Targher *et al.* [102] reported that decreased vitamin D (as 25-hydroxyvitamin D) concentrations in patients with biopsy-proven NAFLD were significantly (*p* < 0.001) and independently associated with increased histological severity of hepatic steatosis and fibrosis. Sprague-Dawley rats fed a high-fat/high-fructose corn syrup diet deficient in vitamin D showed greater hepatic steatosis and up-regulated gene expression of markers of oxidative stress and inflammation, compared to the group with sufficient vitamin D [103]. However, there is insufficient evidence to recommend that patients with NAFLD consume more than the RDA for vitamin D (600 IU/day for 19–70 year old males and females, and 800 IU/day for adults over 70 years). Future investigations should examine the biological role of these vitamins in NAFLD pathogenesis, and whether increasing their amount through diet or supplementation to that beyond the RDA has therapeutic effects.

### 4.5. Summary of Medical Nutrition Therapy

There are limited randomized intervention studies to generate evidence-based dietary recommendations for NAFLD. Carbohydrate source, fiber, and glycemic index are important to consider in meal planning for NAFLD patients. The ADA recommends consuming a diet rich in carbohydrate from fruits, vegetables, whole grains, and legumes for good health and improved blood glucose control. A moderate-carbohydrate diet (40%–65% of energy) is consistent with the ADA recommendations [8,10] and DGAs (Table 2) [49].

Patients with NAFLD may benefit from a moderate- to low-carbohydrate (40%–45% of total calories) diet, coupled with increased dietary MUFA and *n*-3 PUFAs. This macronutrient distribution is typical of Mediterranean-style diets. As highlighted by McCarthy and Rinella [47], avoiding HFCS may be an important recommendation for patients with NAFLD. Diets rich in *n*-3 PUFAs and MUFAs may confer additional anti-inflammatory and cardiovascular benefits, particularly when they replace SFAs [47]. Either low-carbohydrate or low-fat, calorie-restricted diets may be effective in the short term (up to 1-year), and nutrition counseling should be individually based. At this time, there is insufficient evidence for or against the use of nutritional supplements in the NAFLD population, with or without insulin resistance, but patients with NASH may benefit from antioxidant supplementation with vitamin E (800 IU/day).

## 5. Conclusions

This review highlights the growing prevalence of NAFLD. NAFLD is regarded as the hepatic manifestation of the metabolic syndrome and shares common comorbidities with insulin resistance and CVD. It is important for clinicians to recognize this patient population and deliver effective therapeutic lifestyle interventions. In addition, patients may benefit from referral to MNT to optimize dietary intake and promote behavioral changes [104]. The ADA provides evidence-based nutrition recommendations [8,9,10] for preventing and managing type 2 diabetes, insulin resistance, and reducing CVD risk factors. We propose these guidelines are complementary to existing guidelines for NAFLD [7], and can serve as a facilitator in the development of evidenced-based lifestyle intervention recommendations. Future research is needed to evaluate low-carbohydrate/high MUFA and low-fat/low-GI diets for NAFLD. Challenges in evaluating the evidence regarding the effects of foods and macronutrients on metabolic parameters in NAFLD management include variability in study methodology (e.g., measurement of dietary intake, small study samples for intervention trials, confounding by weight loss). Additionally, there is abundant literature examining the effects of lifestyle intervention on metabolic parameters such as lipids, glycemic control, and insulin resistance, but few have measured liver-related outcomes. If future studies in the diabetic population consider including NAFLD outcomes (e.g., serum AST/ALT, imaging, biopsy) when feasible, this would greatly add to the literature and facilitate the formation of evidence-based guidelines. It is likely that many patients with diabetes have NAFLD and therefore should follow dietary and lifestyle recommendations provided by the ADA [8,9,10] to achieve and maintain health. The ADA recommendations [8,9,10] may also benefit non-diabetic NAFLD patients by promoting weight management and preventing the onset of additional metabolic complications.
